# Family functioning types and self-injury risk among left-behind secondary school students: a latent profile analysis with shame as a mediator

**DOI:** 10.3389/fpsyg.2026.1799872

**Published:** 2026-05-18

**Authors:** Cuixiao Wang, Juan Li, Zhanqun Gao, Jianping Wen, Wei Mi

**Affiliations:** 1 Hunan University of Medicine General Hospital, Huaihua, Hunan, China; 2 Huaihua Hongyu Middle School, Huaihua, China; 3 School of Nursing, Hunan University of Medicine, Huaihua, China

**Keywords:** family functioning, latent profile analysis, left-behind secondary school students, non-suicidal self-injury, shame

## Abstract

**Objective:**

To identify latent profiles of family functioning among left-behind secondary school students and to examine their associations with non-suicidal self-injury (NSSI), as well as the mediating role of shame.

**Methods:**

A cross-sectional survey using cluster sampling was conducted in 2024 among 2,385 secondary school students from two rural schools in Huaihua City, Hunan Province. Family functioning was assessed using the Family APGAR scale, shame was measured using the Shame Scale, and non-suicidal self-injury (NSSI) was evaluated based on DSM-5 criteria via a self-report questionnaire. Latent profile analysis (LPA) was used to classify family functioning. Multivariable logistic regression and linear regression models were applied to assess the associations among family functioning, shame, and NSSI. Causal mediation analysis was performed to evaluate the mediating effect of shame.

**Results:**

Three latent profiles of family functioning were identified: low, moderate, and high functioning. After adjusting for potential confounders: (1) compared with students in the high-functioning profile, those in the low-functioning (OR = 1.686, 95% CI: 1.346–2.114) and moderate-functioning profiles (OR = 2.880, 95% CI: 2.125–3.921) had a significantly higher risk of NSSI; (2) shame scores were significantly higher in the low-functioning (β = 2.987) and moderate-functioning profiles (β = 3.762) (both P < 0.001); and (3) each one-point increase in shame was associated with a 33% higher risk of NSSI (OR = 1.330, 95% CI: 1.260–2.041). Mediation analyses indicated that shame partially mediated the association between family functioning and NSSI (low vs. high functioning: mediated proportion = 17.20%; moderate vs. high functioning: mediated proportion = 10.32%).

**Conclusion:**

Family functioning among left-behind secondary school students is heterogeneous. Lower levels of family functioning not only directly increase the risk of NSSI but also indirectly influence self-injurious behavior through elevated shame. Shame represents a key psychological mechanism. These findings suggest that interventions should focus on improving family functioning and reducing shame, such as enhancing family communication and providing school-based psychological support, to effectively prevent and reduce NSSI among this vulnerable population.

## 1 Introduction

With the rapid advancement of urbanization in China, a substantial proportion of the rural labor force has migrated to urban areas for employment, resulting in a large population of left-behind children. Left-behind children span a wide range of developmental stages; however, increasing attention has been focused on mental health problems during secondary school, a critical period for psychological development (Yang et al., 2025). Left-behind secondary school students are typically cared for by grandparents or other guardians and experience prolonged separation from their parents, often accompanied by insufficient emotional support and supervision, which exposes them to multiple psychological and behavioral risks during adolescence (Gao et al., 2024). Previous studies have demonstrated that left-behind experiences are closely associated with a range of mental health problems among adolescents, including depression, anxiety, low self-esteem, and behavioral difficulties (Zheng et al., 2025). In addition, reduced emotional support and diminished quality of family supervision further increase the risk of developmental problems, particularly among rural secondary school students.

Non-suicidal self-injury (NSSI) represents a prevalent psychological and behavioral problem during adolescence, with a significantly higher prevalence reported among left-behind secondary school students than among their non-left-behind peers (Yong et al., 2023). According to the Diagnostic and Statistical Manual of Mental Disorders, Fifth Edition (DSM-5), NSSI refers to the deliberate and direct destruction of one’s own body tissue without suicidal intent and typically includes behaviors such as cutting, hitting, burning, or stabbing (Iswanti et al., 2024). Adolescence is a critical period for the development of emotional regulation and psychological adaptation, and experiences of parental absence during this stage may further increase vulnerability to self-injurious behaviors, thereby exerting long-term adverse effects on mental health and social adjustment.

Family functioning is widely recognized as an important protective factor for adolescent mental health and encompasses multiple dimensions, including emotional expression, communication, support and care, conflict resolution, and overall family satisfaction (Fernandez et al., 2024). Prior research has consistently shown that adolescents from families with poorer functioning are more likely to experience emotional disorders and engage in self-injurious behaviors, whereas those from well-functioning families tend to demonstrate greater psychological resilience and more adaptive coping strategies (Yang et al., 2025). For left-behind secondary school students, long-term parental migration may compromise family functioning through reduced emotional support, impaired communication, and weakened family cohesion, thereby increasing the risk of psychological and behavioral problems (Xue et al., 2026). Shame, a negative self-conscious emotion characterized by global negative self-evaluation, has been increasingly recognized as a key factor in psychological stress and self-injurious behaviors (Park et al., 2021). Levels of shame are strongly influenced by the family environment, parent–child relationships, and social support. Individuals with elevated levels of shame may engage in NSSI as a maladaptive strategy to regulate internal distress or express overwhelming emotions; therefore, shame may serve as an important psychological mechanism linking family functioning to self-injurious behavior (Mürner-Lavanchy et al., 2022). Taken together, these findings suggest that shame may serve as a potential psychological mechanism linking family functioning to NSSI. Therefore, it is reasonable to hypothesize that shame may mediate the association between family functioning and non-suicidal self-injury among left-behind adolescents.

Despite growing interest in the roles of family functioning and shame in adolescent mental health, most existing studies have relied on total scores or single-dimension analyses, which limit their ability to adequately capture the heterogeneity of family functioning. Latent profile analysis (LPA) enables individuals to be classified into distinct subgroups based on multidimensional patterns of family functioning, thereby identifying latent family profiles that may provide more precise insights into psychological and behavioral risks. Moreover, studies examining the role of shame in the association between family functioning and self-injury have primarily adopted cross-sectional correlational designs, offering limited evidence regarding underlying psychological mechanisms. Moreover, adolescents from different family functioning patterns may exhibit distinct psychological characteristics and risk profiles, which cannot be adequately identified using traditional variable-centered approaches. Therefore, adopting a person-centered approach to examine heterogeneous subgroups of family functioning is necessary to better understand differential risk pathways and to inform targeted interventions (Xue et al., 2025).

Therefore, this study integrates latent profile analysis with causal mediation modeling to identify heterogeneous profiles of family functioning among left-behind secondary school students and to quantitatively examine the mediating role of shame in the association between specific family functioning profiles and NSSI. We hypothesized that: (1) distinct latent profiles of family functioning exist among left-behind secondary school students; (2) compared with students from high-functioning families, those from lower-functioning family profiles have a higher risk of NSSI; and (3) shame mediates the association between family functioning and NSSI. By elucidating the psychological mechanisms linking the family environment to self-injurious behavior, this study may inform school-, family-, and public health–based interventions by identifying high-risk subgroups and potential psychological targets for prevention and intervention.

## 2 Materials and methods

### 2.1 Study design and participants

This study employed a cross-sectional design and used cluster sampling to recruit participants from two rural schools in Huaihua City, Hunan Province, China, between March and July 2024. Data were collected on-site by a trained research team, who provided standardized instructions to ensure that students fully understood the study objectives and participated voluntarily.

The inclusion criteria were as follows: (1) students enrolled in secondary school (Grades 7 through 12) and (2) the ability to fully comprehend the questionnaire items. Students who declined to participate were excluded. The study protocol was approved by the school ethics committee [Approval No. Huyi Ethics Review 2025(H03009)], and informed consent was obtained from all participating students.

### 2.2 Measures

Sociodemographic characteristics were collected using a self-designed questionnaire and included age, sex, grade, only-child status, average annual household income, type of left-behind status, and parental educational level. These variables were treated as covariates in subsequent analyses.

#### 2.2.1 Non-suicidal self-injury (NSSI)

NSSI was assessed based on the Diagnostic and Statistical Manual of Mental Disorders, Fifth Edition (DSM-5), using a self-report questionnaire to evaluate the occurrence of self-injurious behaviors in the past 12 months (e.g., burning, cutting, hitting, or stabbing) without suicidal intent. NSSI was initially assessed using a binary response (yes/no). The scale comprised 17 specific self-injurious behaviors, with severity scores calculated by weighting both the frequency of occurrence (0 = 0 times, 1 = 1 time, 2 = 2–4 times, 3 = ≥ 5 times) and the degree of injury (0 = none, 1 = mild, 2 = moderate, 3 = severe, 4 = extremely severe). The scale demonstrated good internal consistency (Cronbach’s α = 0.85) and validity. A total score greater than 0 was considered indicative of NSSI (Sekowski et al., 2020). In subsequent analyses, NSSI was treated as a binary outcome variable.

#### 2.2.2 Shame

Shame was measured using a self-report scale developed by Qi Shenghua (2006/2008), consisting of 22 items across four dimensions: behavioral shame, characterological shame, bodily shame, and competence-related shame. Items were rated on a 4-point Likert scale ranging from 1 (never) to 4 (often), with higher total scores indicating higher levels of shame. The scale showed excellent internal consistency (Cronbach’s α = 0.93) and good test–retest reliability (0.87) (Wang et al., 2025).

#### 2.2.3 Family functioning

Family functioning was assessed using the Family APGAR (Adaptation, Partnership, Growth, Affection, Resolve) scale, which includes six items rated on a 3-point scale (0 = hardly ever, 2 = almost always), with higher scores indicating healthier family functioning. The scale demonstrated high internal consistency (Cronbach’s α = 0.94) and acceptable test–retest reliability (0.80–0.83). Item-level scores were used to conduct latent profile analysis (LPA) to identify distinct family functioning profiles (Griffin et al., 2021).

### 2.3 Statistical analysis

Descriptive analyses included all surveyed participants. Categorical variables were summarized as frequencies and percentages [n (%)], whereas continuous variables were presented as the mean ± standard deviation (SD) or median (interquartile range), depending on their distribution. Group differences according to NSSI status (yes/no) were examined using chi-square tests for categorical variables and independent-samples *t*-tests or Wilcoxon rank-sum tests for continuous variables, as appropriate.

Family functioning was assessed using six-item scores treated as continuous indicators in latent profile analysis (LPA). Models specifying one to five latent classes were estimated and compared using the log-likelihood, Akaike information criterion (AIC), Bayesian information criterion (BIC), sample-size–adjusted BIC (aBIC), entropy, the Lo–Mendell–Rubin adjusted likelihood ratio test (LMR), and the bootstrap likelihood ratio test (BLRT). The optimal number of latent profiles was determined based on overall model fit and interpretability.

Subsequent regression and mediation analyses were restricted to left-behind secondary school students. Using the high family functioning profile as the reference group, multivariable logistic regression models were applied to examine the associations between family functioning profiles and NSSI, as well as between shame scores and NSSI. Multivariable linear regression models were used to assess the associations between family functioning profiles and shame scores. Crude models, demographic-adjusted models, and fully adjusted models were sequentially constructed, controlling for potential confounders including sex, age, grade, household income, and parental migration–related characteristics.

Mediation analyses examining the relationships among family functioning profiles, shame, and NSSI were conducted using the mediation package in R (version 4.5.1). Indirect effects were estimated using non-parametric bootstrap resampling with 5,000 iterations. The average causal mediation effect (ACME), average direct effect (ADE), total effect, and proportion mediated were reported. Mediation effects were considered statistically significant when the 95% confidence interval (CI) for ACME did not include zero. Sensitivity analyses were performed by treating the total family functioning score as a continuous variable in the mediation models. All statistical analyses and figures were conducted using R (4.5.1) and Mplus (8.3), and a two-sided *P*-value < 0.05 was considered statistically significant. (Note: In reporting results, OR = odds ratio; β = unstandardized regression coefficient; CI = confidence interval.)

## 3 Results

### 3.1 Participant characteristics

A total of 2,385 secondary school students were included in the survey, among whom 1,735 were left-behind secondary school students, covering students from Grade 7 to Grade 12. Overall, 1,267 students reported engaging in non-suicidal self-injury (NSSI), while 1,118 reported no history of NSSI.

Significant differences in the prevalence of NSSI were observed according to left-behind status, age, shame scores, and family functioning scores (*P* < 0.05). In addition, NSSI was more prevalent among male students, junior secondary school students, those from lower-income households, left-behind students, those reporting lower frequency of contact with migrant parents, and those holding negative attitudes toward parental labor migration (Table 1).

**TABLE 1:** Basic characteristics of the study participants (n, %).

Variable	Overall (*n* = 2,385)	With NSSI (*n* = 1,267)	Without NSSI (*n* = 1,118)	*P-*value
Sex				
Male	1242 (52.1)	711 (56.1)	531 (47.5)	< 0.001
Female	1143 (47.9)	556 (43.9)	587 (52.5)	
Age [mean (SD)]	14.79 (1.44)	15.02 (1.50)	14.53 (1.33)	< 0.001
Grade				
7	421 (17.7)	185 (14.6)	236 (21.1)	< 0.001
8	644 (27.0)	292 (23.0)	352 (31.5)	
9	591 (24.8)	299 (23.6)	292 (26.1)	
10	359 (15.1)	226 (17.8)	133 (11.9)	
11	273 (11.4)	198 (15.6)	75 (6.7)	
12	97 (4.1)	67 (5.3)	30 (2.7)	
Only-child status				
Yes	320 (13.4)	169 (13.3)	151 (13.5)	0.952
No	2065 (86.6)	1098 (86.7)	967 (86.5)	
Annual family income				
< 20,000 yuan	722 (30.3)	375 (29.6)	347 (31.0)	0.001
20,000–50,000 yuan	831 (34.8)	411 (32.4)	420 (37.6)	
50,000–80,000 yuan	533 (22.3)	297 (23.4)	236 (21.1)	
80,000–100,000 yuan	289 (12.1)	181 (14.3)	108 (9.7)	
> 100,000 yuan	10 (0.4)	3 (0.2)	7 (0.6)	
Father’s occupation				
Farming	351 (14.7)	201 (15.9)	150 (13.4)	0.307
Migrant worker	898 (37.7)	460 (36.3)	438 (39.2)	
Government/Public sector employee (e.g., civil servant, teacher, doctor)	126 (5.3)	69 (5.4)	57 (5.1)	
Self-employed/Business	364 (15.3)	201 (15.9)	163 (14.6)	
Other	646 (27.1)	336 (26.5)	310 (27.7)	
Mother’s occupation				
Farming	462 (19.4)	249 (19.7)	213 (19.1)	0.165
Migrant worker	628 (26.3)	352 (27.8)	276 (24.7)	
Government/Public sector employee (e.g., civil servant, teacher, doctor)	121 (5.1)	56 4.4)	65 (5.8)	
Self-employed/Business	389 (16.3)	211 (16.7)	178 (15.9)	
Other	784 (32.9)	398 (31.4)	386 (34.5)	
Father’s educational level				
Junior secondary school or below	1638 (68.7)	855 (67.5)	783 (70.0)	0.196
Senior secondary school or vocational secondary school	601 (25.2)	338 (26.7)	263 (23.5)	
College or above	146 (6.1)	74 (5.8)	72 (6.4)	
Mother’s educational level				
Junior secondary school or below	1775 (74.5)	947 (74.8)	828 (74.1)	0.55
Senior secondary school or vocational secondary school	509 (21.4)	262 (20.7)	247 (22.1)	
College or above	100 (4.2)	57 (4.5)	43 (3.8)	
Left-behind experience				
Yes	1735 (72.7)	891 (70.3)	844 (75.5)	0.005
No	650 (27.3)	376 (29.7)	274 (24.5)	
Shame score [mean (SD)]	22.94 (15.56)	19.03 (14.08)	27.37 (15.97)	< 0.001
Family functioning score [mean (SD)]	14.88 (6.75)	16.11 (6.60)	13.48 (6.66)	< 0.001

### 3.2 Latent profile analysis of family functioning

Latent profile analysis (LPA) was conducted among the 1,735 left-behind secondary school students to identify distinct patterns of family functioning. Models specifying one to five latent profiles were estimated, and the corresponding model fit indices are presented in Table 2. As the number of latent profiles increased, the log-likelihood values increased progressively, while the AIC, BIC, and sample-size adjusted BIC (aBIC) values generally decreased. The most pronounced improvement in model fit was observed when moving from the two-profile to the three-profile solution, indicating a substantial enhancement in model performance.

**TABLE 2:** Model fit indices for latent profile analysis (LPA) of family functioning (*n* = 1,735).

Model	Log(L)	AIC	BIC	aBIC	Entropy	LMR (P)	BLRT (P)	Class proportions
1-class	−23767.567	47559.135	47628.478	47590.352	–	–	–	1
2-class	−19968.099	39974.198	40083.991	40023.624	0.903	< 0.001	<0.001	0.453, 0.547
3-class	−17971.086	35994.171	36144.416	36061.808	0.925	< 0.001	<0.001	0.410, 0.161, 0.429
4-class	−17172.915	34411.829	34602.524	34497.676	0.919	< 0.001	<0.001	0.322, 0.150, 0.329, 0.198
5-class	−17063.819	34207.639	34438.784	34311.695	0.908	0.3322	0.0000	0.1515, 0.2951, 0.3135, 0.1963, 0.0435

The three-profile model demonstrated relatively high entropy, suggesting good classification accuracy, and both the Lo–Mendell–Rubin adjusted likelihood ratio test (LMR) and the bootstrap likelihood ratio test (BLRT) were statistically significant (*P* < 0.001). Although the four-profile model showed further improvement in some fit indices, the gains in classification accuracy were limited, and model complexity increased. Considering overall model fit, classification precision, and interpretability, the three-profile solution was selected as the optimal model.

**FIGURE 1:**
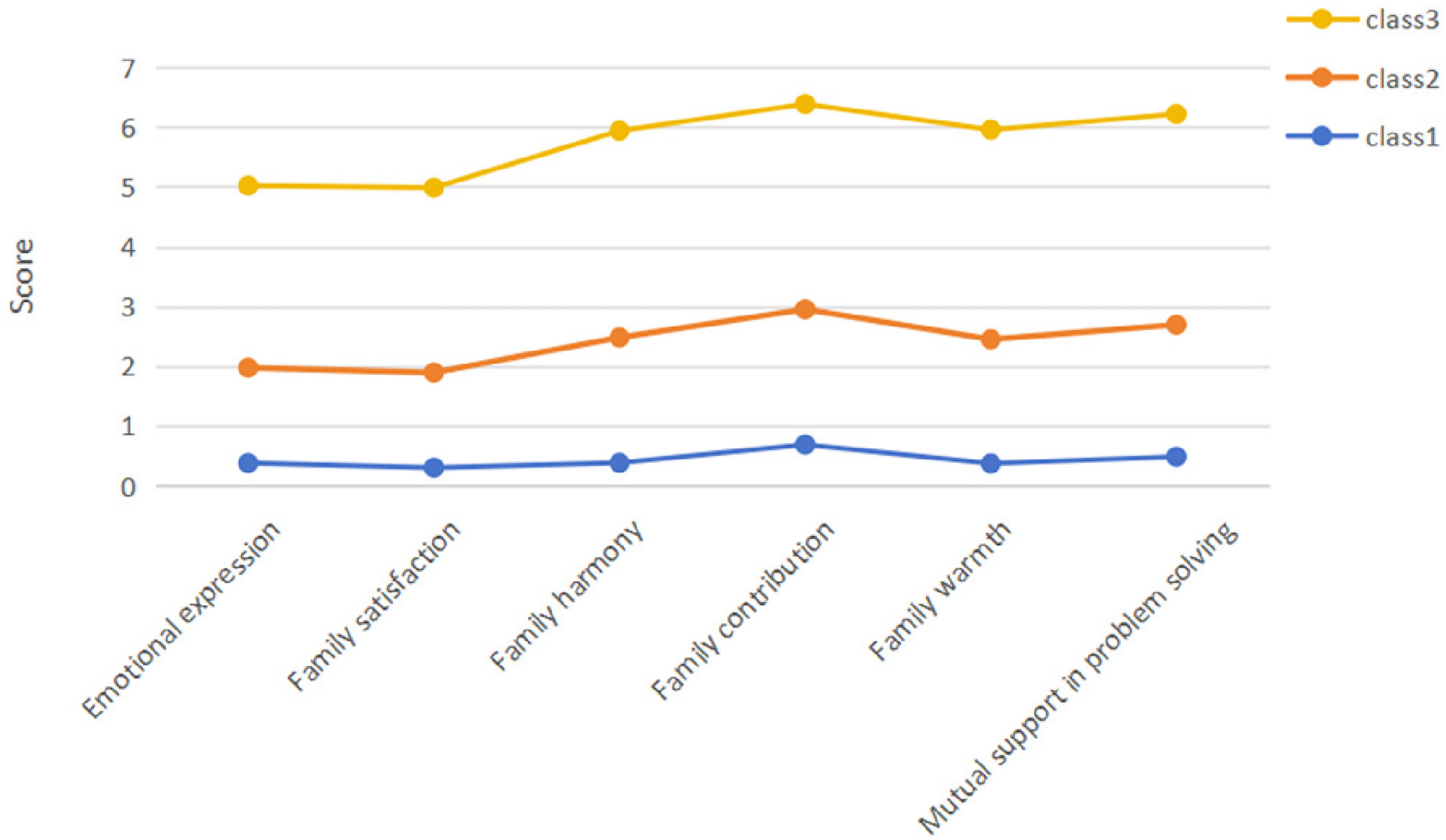
Family functioning profiles among left-behind secondary school students based on latent profile analysis (LPA). Class 1 = low-functioning family class; Class 2 = moderate-functioning family class; Class 3 = high-functioning family class.

Comparisons of the three latent profiles across the six dimensions of family functioning are illustrated in Figure 1, revealing clear differentiation among profiles. Profile 1 exhibited consistently low scores across emotional expression, family satisfaction, family harmony, family contribution, family warmth, and mutual support in problem-solving, and was therefore labeled the low family functioning profile. Profile 2 demonstrated moderate scores across these dimensions, reflecting a certain level of emotional interaction and support, and was labeled the moderate family functioning profile. Profile 3 showed significantly higher scores across all family functioning dimensions compared with the other two profiles, indicating strong family cohesion and supportive characteristics, and was labeled the high family functioning profile.

### 3.3 Association between family functioning classes and non-suicidal self-injury

Multivariable logistic regression models were used to examine the associations between different latent classes of family functioning and non-suicidal self-injury (NSSI), with the high-functioning family class as the reference. Three models were constructed: unadjusted (Model 1), demographic-adjusted (Model 2), and fully adjusted (Model 3). Across all models, significant associations were observed between latent classes of family functioning and NSSI. Compared with the high-functioning family class, adolescents in the low- and moderate-functioning family classes exhibited a higher risk of NSSI in all models.

**FIGURE 2:**
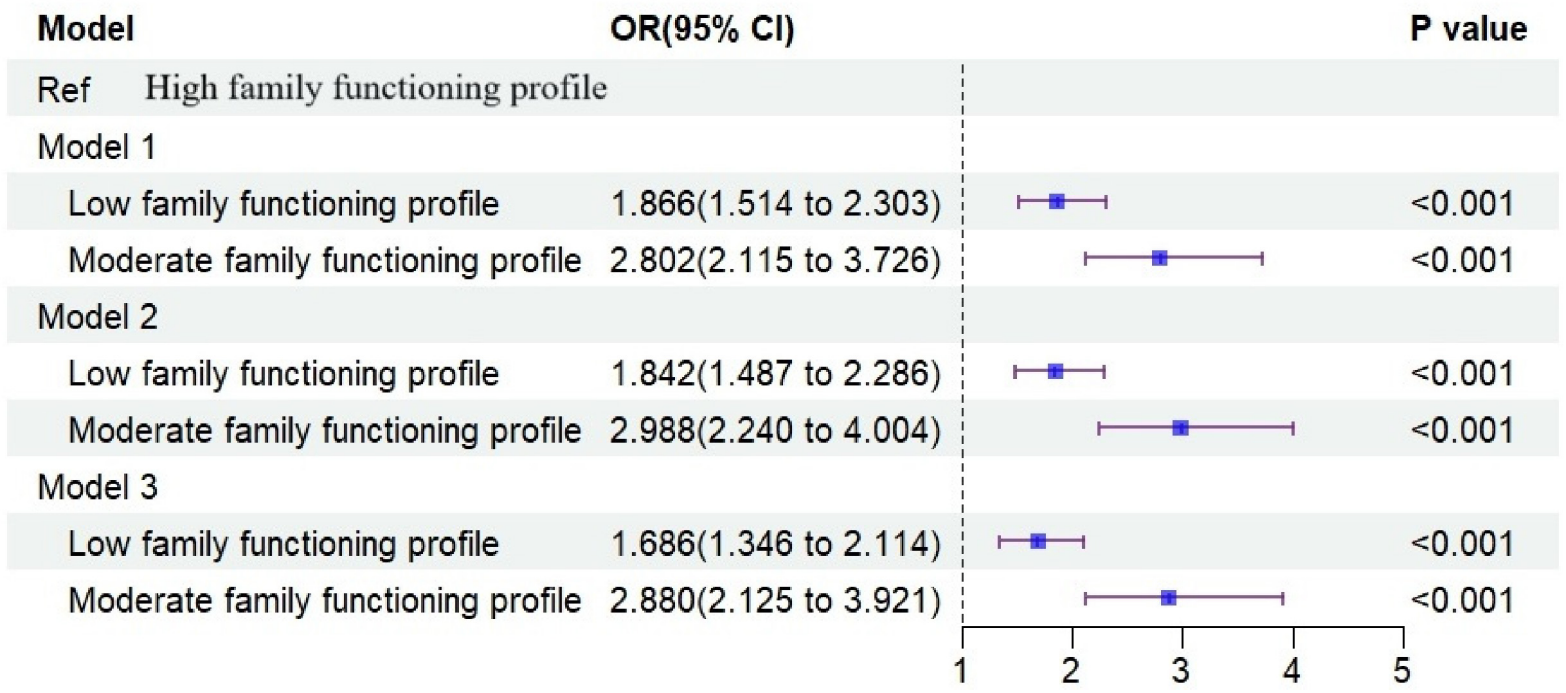
Forest plot of multivariable logistic regression analyses examining the associations between latent classes of family functioning and NSSI among left-behind secondary school students.

In the fully adjusted model, after controlling for potential confounders, adolescents in the low-functioning family class had a significantly increased risk of NSSI (OR = 1.686, 95% CI: 1.346–2.114, *P* < 0.001), while those in the moderate-functioning family class had an even higher risk (OR = 2.880, 95% CI: 2.125–3.921, *P* < 0.001) (Figure 2). These findings indicate that lower family functioning remains significantly associated with NSSI even after comprehensive adjustment for covariates.

### 3.4 Association between family functioning classes and shame

Multivariable linear regression models were used to investigate the associations between latent classes of family functioning and shame scores, with the high-functioning family class as the reference. Three models were constructed: unadjusted (Model 1), demographic-adjusted (Model 2), and fully adjusted (Model 3). Significant associations were observed across all models, with adolescents in the low- and moderate-functioning family classes showing significantly higher shame scores compared with those in the high-functioning family class (all *P* < 0.001), and the direction of effects was consistent (Table 3).

**TABLE 3:** Associations between latent classes of family functioning and shame scores among left-behind secondary school students.

Model	*P*-value	Estimate	StdError
Ref	High family functioning profile		
Model 1			
Low family functioning profile	< 0.001	4.194	0.81
Moderate family functioning profile	< 0.001	4.825	1.074
Model 2			
Low family functioning profile	< 0.001	3.536	0.792
Moderate family functioning profile	< 0.001	4.273	1.047
Model 3			
Low family functioning profile	< 0.001	2.987	0.811
Moderate family functioning profile	< 0.001	3.762	1.074

In the fully adjusted model (Model 3), after further controlling for sex, age, grade, only-child status, annual family income, parental education level, and parental migration-related characteristics, the associations between family functioning classes and shame scores remained significant. Compared with the high-functioning family class, adolescents in the low-functioning class had an average increase of 2.99 points in shame scores (β = 2.987, SE = 0.811, *P* < 0.001), and those in the moderate-functioning class had an average increase of 3.76 points (β = 3.762, SE = 1.074, *P* < 0.001) (Table 3).

### 3.5 Association between shame and non-suicidal self-injury

**FIGURE 3:**
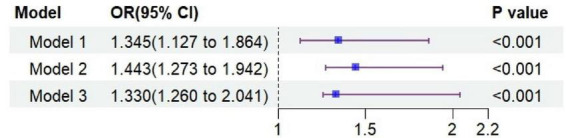
Forest plot of logistic regression analyses examining the association between shame scores and NSSI among left-behind secondary school students.

Logistic regression models were applied to examine the association between shame scores and NSSI, using unadjusted (Model 1), demographic-adjusted (Model 2), and fully adjusted (Model 3) models. In all models, shame scores were significantly positively associated with NSSI. In the fully adjusted model (Model 3), after further controlling for grade, only-child status, annual family income, parental education level, parental migration characteristics, and adolescents’ attitudes toward parental migration, higher shame scores remained significantly associated with increased risk of NSSI. Specifically, each one-point increase in shame score was associated with approximately a 33% higher risk of NSSI (OR = 1.330, 95% CI: 1.260–2.041, *P* < 0.001) (Figure 3).

### 3.6 Mediating role of shame in the association between family functioning classes and non-suicidal self-injury

Mediation analyses were conducted to examine whether shame mediated the relationship between latent classes of family functioning and non-suicidal self-injury (NSSI). The high-functioning family class (Class 3) was used as the reference group, and comparisons were made with the low-functioning (Class 1) and moderate-functioning (Class 2) classes. Three models were sequentially constructed: unadjusted, demographic-adjusted (adjusting for sex and age), and fully adjusted.

#### 3.6.1 Comparison of class 1 (low-functioning) and class 3 (high-functioning)

**FIGURE 4:**
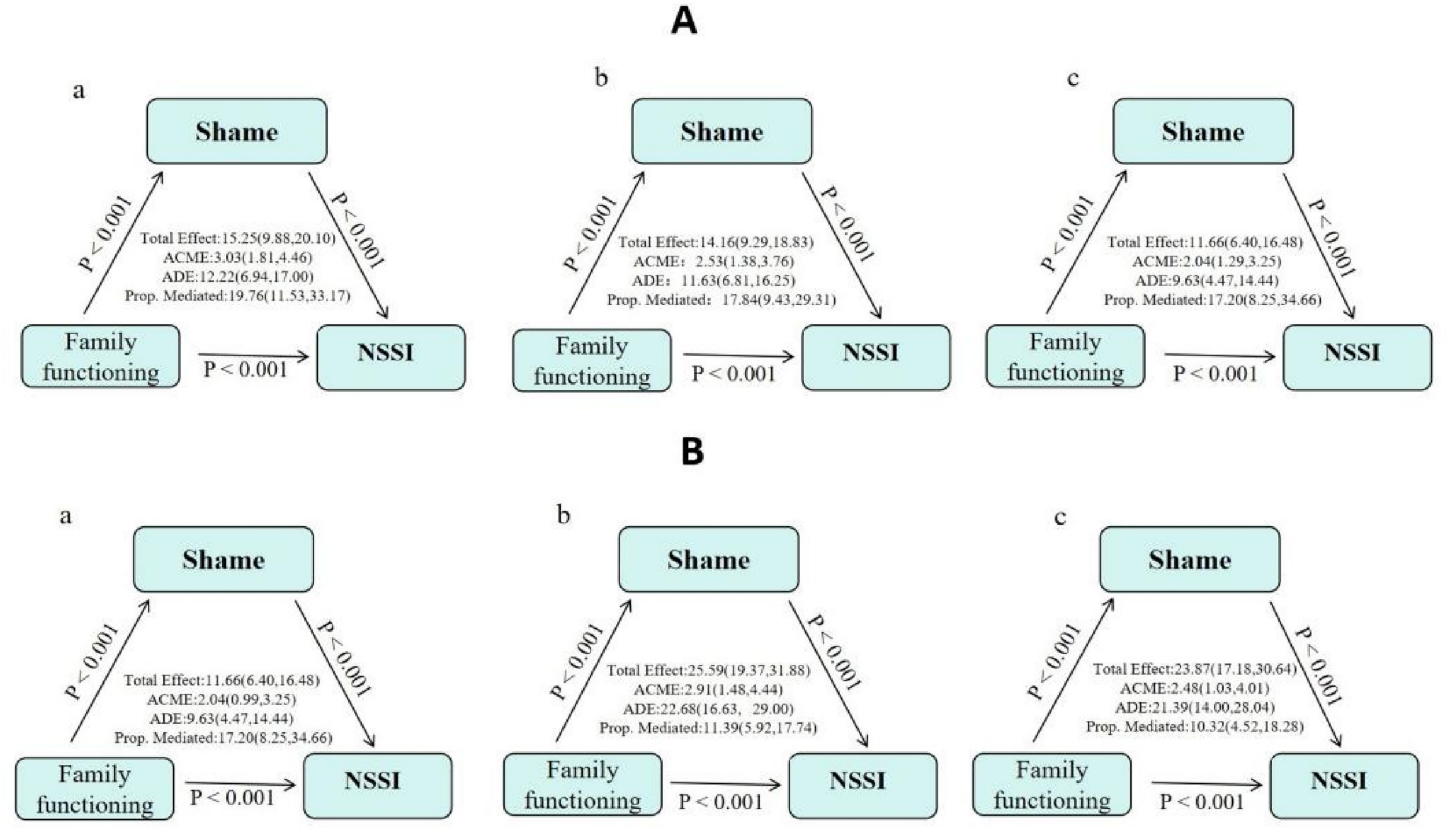
Mediation models illustrate the role of shame in the association between latent classes of family functioning and non-suicidal self-injury (NSSI). **(A)** Low-functioning family class vs. high-functioning family class. **(B)** Moderate-functioning family class vs. high-functioning family class. Models: (a) unadjusted model; (b) partially adjusted model (adjusted for sex and age); (c) fully adjusted model. ACME, average causal mediation effect; ADE, average direct effect.

Across all three models, shame was found to significantly mediate the association between the low-functioning family class and NSSI. In the fully adjusted model, after further controlling for grade, only-child status, annual family income, parental education, parental migration-related characteristics, and adolescents’ attitudes toward parental migration, the mediating effect of shame remained significant. The total effect of the low-functioning class on NSSI was 11.66% (95% CI: 6.40–16.48%), with an average causal mediation effect (ACME) of 2.04% (95% CI: 1.29–3.25%), an average direct effect (ADE) of 9.63% (95% CI: 4.47–14.44%), and a proportion mediated of 17.20% (95% CI: 8.25–34.66%) (Figure 4).

#### 3.6.2 Comparison of class 2 (moderate-functioning) and class 3 (high-functioning)

Similarly, in all three models, shame significantly mediated the association between the moderate-functioning family class and NSSI. In the fully adjusted model, after controlling for all covariates, the total effect of the moderate-functioning class on NSSI was 23.87% (95% CI: 17.18–30.64%), with an ACME of 2.48% (95% CI: 1.03–4.01%), an ADE of 21.39% (95% CI: 14.00–28.04%), and a proportion mediated of 10.32% (95% CI: 4.52–18.28%) (Figure 4).

Overall, shame partially mediated the relationship between latent classes of family functioning and NSSI in both low- and moderate-functioning family classes. This mediating effect remained stable across different levels of adjustment.

### 3.7 Sensitivity analysis

To assess the robustness of the findings, family functioning scores were additionally treated as a continuous variable in the mediation models. Unadjusted and fully adjusted models were constructed. In the fully adjusted model, family functioning scores were significantly negatively associated with NSSI, with shame partially mediating this relationship (proportion mediated = 9.53%, 95% CI: 4.05–16.34%). In the unadjusted model, the direction and significance of the results were consistent with those of the fully adjusted model (Supplementary Figure 1).

## 4 Discussion

This study applied latent profile analysis to identify latent classes of family functioning among left-behind secondary school students and examined the associations between different family functioning classes and non-suicidal self-injury (NSSI), while testing the mediating role of shame. The findings indicated that family functioning among left-behind secondary school students could be categorized into three latent classes: low, moderate, and high. Students in the low- and moderate-functioning classes had significantly higher risks of engaging in NSSI compared with those in the high-functioning class, and shame partially mediated the relationship between family functioning and NSSI. These results were robust across different adjustment models and were further supported by sensitivity analyses.

The findings of this study reflect the mental health characteristics of left-behind secondary school students. Being left behind is often accompanied by prolonged parental absence due to labor migration, resulting in a lack of continuous parental companionship and emotional support during critical developmental periods. Family structures characterized by caregiving from grandparents or other guardians may be associated with limited family functioning. Low family functioning is typically manifested by insufficient emotional expression, reduced family support and cohesion, and limited conflict resolution capacity (Taylor et al., 2018), which can increase adolescents’ psychological stress and emotional distress, thereby elevating the risk of NSSI. In contexts of restricted family functioning, adolescents may struggle to obtain stable emotional support and a sense of security, and negative emotions may not be recognized or effectively addressed in a timely manner (Shi et al., 2026). Prolonged exposure to such environments may lead individuals to adopt internalized and suppressed coping patterns rather than seeking open communication or external support to alleviate psychological distress. Furthermore, low family functioning may weaken adolescents’ psychological resilience to stress, making them more likely to engage in maladaptive behaviors such as NSSI when facing academic, interpersonal, or self-development pressures (Wan et al., 2025). Consistent with theoretical expectations, the prevalence of NSSI was significantly higher among students in the low- and moderate-functioning classes than among those in the high-functioning class, supporting the view that family functioning serves as a protective factor. These findings are also in line with previous research demonstrating close associations between poor family functioning, adolescent emotional disorders, and self-injurious behaviors (Zhao et al., 2023). Importantly, the use of latent profile analysis revealed heterogeneity in family functioning that cannot be captured by traditional total-score or unidimensional approaches, thereby enabling more precise identification of high-risk subgroups and providing clearer targets for psychological intervention.

Mediation analyses demonstrated that shame played a significant role in the pathway linking family functioning to NSSI in both the low- and moderate-functioning classes. This finding is consistent with psychological theories suggesting that individuals with elevated levels of shame may lack effective emotion regulation strategies when encountering emotional stress, increasing the likelihood of adopting NSSI as a maladaptive coping mechanism (Liu et al., 2022). It is also consistent with previous studies conducted in China and other countries, indicating that shame may indirectly elevate the risk of self-injury or self-harm by impairing self-esteem and emotion regulation capacity (Li et al., 2025). In this study, shame was confirmed as a key psychological mechanism connecting family functioning and NSSI. Left-behind secondary school students, owing to prolonged parental absence and reduced emotional support, may be particularly vulnerable to negative self-evaluation and feelings of shame, which may, in turn, precipitate NSSI as a means of emotion regulation or internalized stress release. As a self-directed negative emotion, shame is often accompanied by self-denial, feelings of worthlessness, and perceived rejection, which may discourage individuals from seeking external support and further exacerbate internalized emotional distress (Kandar Dewi et al., 2025). Prior research has shown that individuals experiencing high levels of shame are more likely to adopt avoidant or self-punitive coping strategies rather than proactively seeking help (Ma et al., 2025). In this context, NSSI may function as a temporary strategy to alleviate shame and psychological pain, despite its clearly harmful long-term consequences (Yang, 2025). Overall, the findings suggest that family functioning influences NSSI risk among left-behind secondary school students partly through its impact on shame, providing empirical support for this mediating mechanism and offering deeper insight into the psychological processes underlying NSSI (Kudinova et al., 2023). Notably, adolescents in the moderate-functioning family profile showed a higher odds ratio for NSSI compared with those in the low-functioning profile when both were referenced to the high-functioning group. However, as no direct statistical comparison was conducted between the low- and moderate-functioning groups, this difference should be interpreted with caution.

This study has important theoretical and practical implications. From a theoretical perspective, the application of latent profile analysis enabled the identification of distinct family functioning classes, accurately capturing heterogeneity in family environments and providing a refined basis for psychological risk prediction. The findings reinforce the protective role of family functioning in adolescent mental health and validate the mediating role of shame as a critical psychological mechanism. From a practical perspective, the results suggest that schools and families should pay particular attention to left-behind secondary school students from low- and moderate-functioning families. Interventions aimed at enhancing family communication, strengthening emotional support, and addressing shame may be effective in reducing the risk of NSSI. Potential strategies include school–family collaborative psychological counseling, maintaining emotional contact during parental migration, and implementing shame-focused emotion regulation training. Nevertheless, several limitations should be noted. First, the cross-sectional design precludes causal inference. Second, the sample was drawn from only two rural schools, which may limit generalizability. Third, the reliance on self-reported data may introduce reporting bias. Future research should employ longitudinal or interventional designs to clarify causal relationships, recruit more diverse samples across regions and populations, and explore additional psychological mechanisms such as depression, anxiety, and self-esteem.

## 5 Conclusion

This study demonstrated significant associations between latent classes of family functioning and non-suicidal self-injury among left-behind secondary school students and confirmed the partial mediating role of shame. The findings highlight two complementary targets for NSSI prevention and intervention: at the environmental level, identifying and supporting families with low or moderate functioning and improving the quality of emotional interactions; and at the individual level, reducing shame and enhancing emotion regulation skills as core components of psychological intervention. Future studies employing longitudinal designs are warranted to further verify this causal pathway and to explore other potential mediating or moderating factors.

## Data Availability

The original contributions presented in the study are included in the article/Supplementary material, further inquiries can be directed to the corresponding author.
