# Supplementary figures and images for: Family functioning types and self-injury risk among left-behind secondary school students: a latent profile analysis with shame as a mediator
Source: Front Psychol. 2026 May 18;17:1799872. doi: 10.3389/fpsyg.2026.1799872 (PMC13222795; doi:10.3389/fpsyg.2026.1799872)

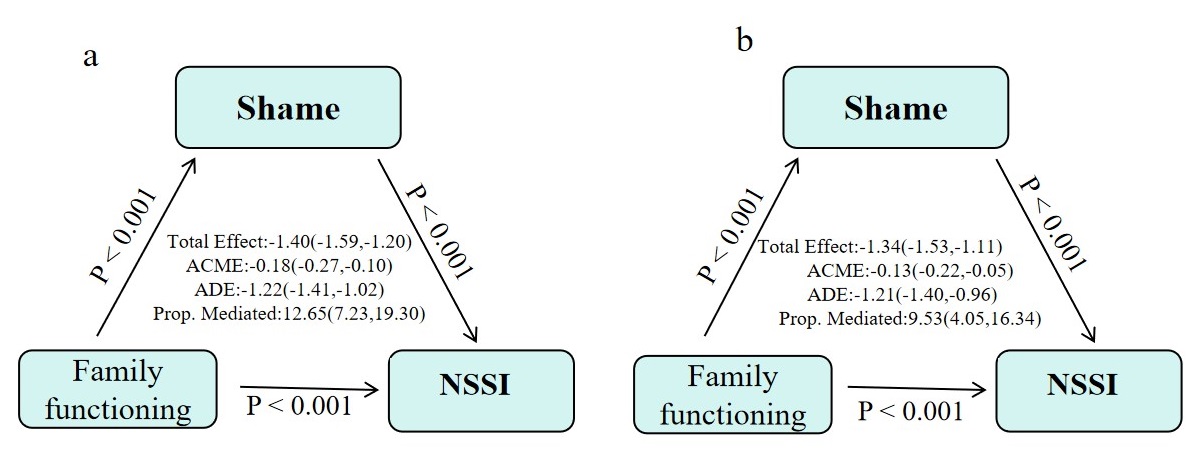

Supplement: Supplementary file 1 [file Image_1.jpeg]
